# Slow-release carbohydrates: growing evidence on metabolic responses and public health interest. Summary of the symposium held at the 12th European Nutrition Conference (FENS 2015)

**DOI:** 10.3402/fnr.v60.31662

**Published:** 2016-07-04

**Authors:** Sophie Vinoy, Martine Laville, Edith J M Feskens

**Affiliations:** 1 Nutrition Department, Mondelez Int R&D, Saclay, France; 2 Department of Nutrition, CRNH-RA, Lyon 1 University, Hospices civils de Lyon, Chemin du grand revoyet Pierre bénite, France; 3 Division of Human Nutrition, Wageningen University, Wageningen, The Netherlands

**Keywords:** cereal foods, carbohydrate quality, glycaemic index, glycaemic response, stable isotope tracers, slow appearance, starch digestibility, slowly digestible starch, metabolic disease prevention

## Abstract

To draw attention to the necessity of considering differences in the digestibility of carbohydrates, and more specifically of starch, a symposium was held at the 12th European Nutrition Conference (FENS), which took place in Berlin from October 20 to 23, 2015. The purpose of this session was to present the consolidated knowledge and recent advances regarding the relationship between slow-release carbohydrates, metabolic responses, and public health issues. Three main topics were presented: 1) the definition of, sources of, and recognised interest in the glycaemic response to slowly digestible starch (SDS); 2) clinical evidence regarding the physiological effects of slow-release carbohydrates from cereal foods; and 3) interest in reducing the postprandial glycaemic response to help prevent metabolic diseases. Foods with the highest SDS content induce the lowest glycaemic responses, as the starch is protected from gelatinisation during processing. In humans, high-SDS food consumption induces slower glucose release, lower postprandial insulinaemia, and stimulation of gut hormones. Moreover, postprandial hyperglycaemia is an independent risk factor for type two diabetes mellitus (T2DM) and cardiovascular disease (CVD). Therefore, given the plausible aetiologic mechanisms, we argue that postprandial glucose levels are relevant for health and disease and represent a meaningful target for intervention, for example, through dietary factors. This symposium was organised by Mondelez International R&D.

Diets that induce small excursions in postprandial plasma glucose and insulin concentrations are associated with a wide range of health benefits, including improved insulin secretion and sensitivity, and thus enhanced glycaemic control (1–3). Moreover, repeated postmeal high blood glucose levels have been associated with an increased risk of cardiovascular events and type two diabetes mellitus (T2DM) (4). Furthermore, in Western countries, most carbohydrate (CHO) foods undergo processing, which modulates starch digestibility (5). As starch is one of the most important glycaemic CHO components in cereal products, specific steps in the manufacturing process may influence its digestibility (6).

It is generally implicitly assumed that differences in the glycaemic response primarily reflect differences in CHO digestibility, and therefore glucose absorption and the rate of glucose appearance in the peripheral circulation. However, several contradictory results have been reported (7, 8). Characterising more specifically starch, among CHOs, based on its sensitivity to digestive amylase, which converts it into slowly digestible starch (SDS), rapidly digestible starch (RDS), and resistant starch (RS), is of nutritional and physiological significance. This classification reflects the impact on postprandial blood glucose homeostasis and the associated metabolic and endocrine responses. Moreover, tracking the absorption kinetics of dietary CHO-derived glucose through stable isotope labelling of exogenous CHO provides crucial complementary information on the mechanisms underlying variations in postprandial glycaemia.

The goal of the symposium was to share consolidated scientific knowledge and recent advances regarding the relationship between slow-release CHOs, low postprandial glycaemic response, and public health. The session started by defining SDS and the relationship between SDS, starch gelatinisation, and the glycaemic response. Then, the consolidated clinical evidence on the physiological effects of SDS was addressed, including an in-depth discussion of postprandial regulation and dynamic approaches to measuring CHO rates. Finally, the key point of the potential implications of reducing postprandial glycaemia to help prevent the development of metabolic diseases was discussed. The symposium, which was chaired by Professor Martine Laville, concluded with a general discussion involving representatives from both academia and industry. The symposium was held during the 12th European Nutrition Conference (FENS), which took place in Berlin from October 20 to 23, 2015, and was organised by Mondelez International R&D, which has conducted over 20 years of research into starch digestibility and its impact on postprandial metabolic responses.

## Presentation summaries

### Slowly digestible starch: definition, sources, and recognised interest in glycaemic response

Dr. Sophie Vinoy, Mondelez International

Based on recommendations from official international institutions (9), starch should represent the largest component of our daily energy intake (45–55%). Based on the most recently published limits for free sugar intake (10) and a total consumption of 2,000 kcal per day, starch intake should therefore consist of around 130–200 g/day. This is consistent with several national dietary guidelines that consider starchy foods to be key dietary staples (11–14).

Food starch is derived from cereals (e.g. wheat, maize, rice, barley, and buckwheat), tubers (e.g. potatoes and cassava) and legumes (e.g. peas, lentils, kidney beans, and mung beans). Starch is a semi-crystalline material produced by plants that forms roughly spherical granules in plant tissues. The sizes and shapes of the granules depend on the botanical origin, as well as the relative amounts of amylose (a straight chain polymer of α-1,4-linked glucose units) and amylopectin (a branched chain polymer that contains α-1,6-linked glucose units at the branch point and α-1,4 links in the linear regions). Based on these variables, the starch digestibility profile varies greatly in its natural state. In addition, several grains have different starch granule structures, which modulate the hydrolysis parameters (15). For example, a study of 15 starchy foods showed that only 2% of water yam starch was hydrolysed, whereas around 85% of rice starch underwent hydrolysis.

The rate and extent of starch digestion can be measured *in vitro* using a method developed by Englyst et al. (16), which classifies starch into three major fractions: RDS, SDS, and RS. This method has been validated by ring test in six different laboratories. Cereal products with a wide range of SDS values (1–24 g/100 g) were selected to represent the typical range of SDS content in cereal foods. The SDS test repeatability, based on intra-laboratory analyses, was 0.7 g/100 g. The SDS test reproducibility was 0.9 g/100 g, which takes into account the variance of food product analyses and the laboratory effect. Finally, uncertainty in the SDS measurement can be calculated by combining the repeatability and the reproducibility. SDS uncertainty for a triplicate analysis was *u*=1.9 g/100 g, meaning that a result can be expressed as±*u* with 95% confidence. These results demonstrate that this method is reliable for measuring SDS, as it is in the same range as several AOAC-approved methods.

**Fig. 1:**
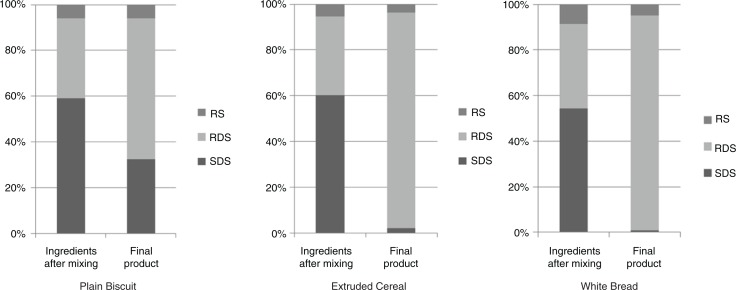
Percent of each starch fraction compared with the total starch content of three different cereal products during food processing. RDS, rapidly digestible starch; RS, resistant starch; SDS: slowly digestible starch. From Cartier (personal communication).

During food manufacturing, heat, moisture, and pressure can dramatically modify the digestibility of starch in processed foods. The combination of high moisture levels and high temperatures (e.g. during baking or drum-drying) or high pressure and shearing (as in extrusion cooking) replaces nearly all of the SDS content with RDS. In contrast, in barley porridge, parboiled rice, biscuits, and pasta, the lower degree of gelatinisation or limited starch swelling, which is mainly determined by moderate moisture levels, cooking time, and temperature, preserves the SDS content (17, 18). To illustrate the impact of these factors, we produced three cereal products that are processed via three different methods: plain biscuits (baking), white bread (bread making), and extruded cereal (baking–extrusion). The SDS content was maintained more effectively in plain biscuits compared with bread and extruded cereal (Fig. 1).

During food processing, the starch granule undergoes dramatic changes when it is heated in the presence of water (19, 20). As the temperature increases, hydrogen bonds between the starch chains are disrupted, and water is absorbed by the starch granule. This leads to swelling of the granule, which is followed by amylose leaching. The starch dissolves progressively, gradually increasing the viscosity of the solution. Gelatinisation leads to the formation of a starch paste (21). There is a clear relationship between SDS and the gelatinisation stage of starch in cereal products (22). Cereal products with the highest levels of SDS had the lowest degree of starch gelatinisation. Thus, controlling food-processing conditions can prevent SDS loss by limiting the extent of starch gelatinisation (22, 23).

**Fig. 2:**
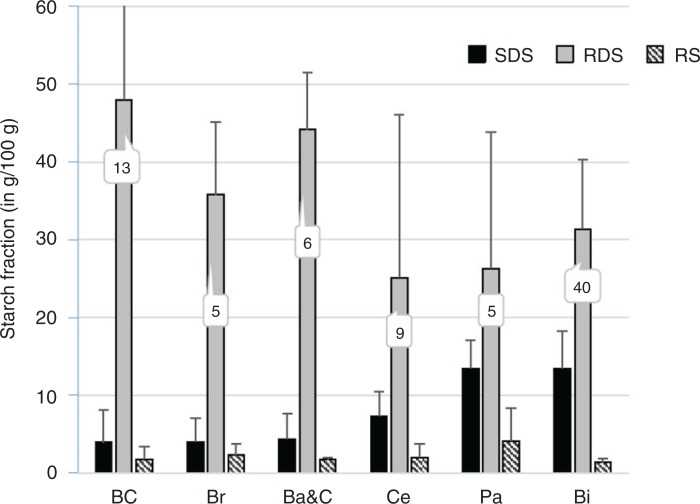
SDS, RDS and RS content of six types of cereal products. BC (breakfast cereals, n=13); Br (bread, n=5); Ba&C (bakery products and crackers, n=6); Ce (cereals, n=9); Pa (pasta, n=5); Bi (biscuits, n=40). RDS, rapidly digestible starch; RS, resistant starch; SDS, slowly digestible starch. From Lintas et al. (24); Englyst et al. (16); Englyst et al. (25); Englyst et al. (22); Garsetti et al. (26).

Furthermore, the combination of different ingredients and food-processing conditions results in a wide range of SDS and RDS content in cereal foods, not only between types of products but also within the same food category (16, 22, 24–26) (Fig. 2).

Preserving starch in its native, slowly digestible form has important health implications. Several studies in humans that compared the physiological effects of starch-based products with different SDS contents have shown a strong link between the *in vitro* digestibility of starch and postprandial plasma glucose and insulin responses (27). Out of 23 cereal foods tested, the products with the highest SDS content and the smallest degree of starch gelatinisation had the lowest glycaemic index (GI), and conversely, cereal foods with the lowest SDS values had the highest GI (22). A recent systematic review of wholegrain oats suggested that the differences in GI values between product types may be due to the different particle size and degree of starch gelatinisation (28). Furthermore, a recent study of 190 cereal products that used an original approach to evaluate the impact of starch digestibility, macronutrient contents, and their interactions on the metabolic response showed that SDS, RDS, fat, fibres, and interactions between these components significantly explained GI by 53%. SDS was the major contributor to GI, and its effect was independent of the content of other macronutrients (29).

To conclude, foods that are rich in SDS due to limiting starch gelatinisation during processing induce the lowest glycaemic responses related to a non-exacerbated insulinaemic response, both of which play a role in preventing the development of metabolic disease.

### Clinical evidence on the physiological effects of slow-release carbohydrates from cereal foods

Prof. M Laville, CRNH-RA, Lyon 1 University

Postprandial glycaemia results from several digestive and/or metabolic regulatory processes that interact with decreasing peripheral blood glucose (27). Moreover, dietary factors such as the amount and type of CHOs ingested (including sugars); the composition, nature, and digestibility of starches; the culinary and technological treatments; and processing or cooking conditions may affect their bioavailability and be a determining factor in the level of postprandial glycaemia. The composition of the concomitant or previous meal and the association of CHOs with lipids or proteins may also have an effect (30, 31). All of these factors can differentially alter the glycaemic response by acting on gastrointestinal factors (such as gastric emptying or intestinal absorption) and hormonal factors (such as insulin, glucagon, gut hormones, or incretins).

When investigating the effect of CHO on glucose absorption, it is important to note that the glycaemic and insulinaemic indexes of foods are peripheral postprandial markers that only partially reflect the absorption kinetics of starch-derived glucose. Moreover, most of these measurements are often limited to 2 h after food consumption, at which time the metabolic processes are far from over. The extraordinary capacity of a healthy human body to adapt to its nutritional intake is well-illustrated by the tight regulation of glucose homeostasis. In the fasting state, glycaemia is maintained within a narrow range due to endogenous glucose production (EGP), primarily by the liver. After glucose is absorbed, it travels to the portal vein and the liver, where it is partly stored in the liver and partly released into the bloodstream, leading to an increase in glycaemia. The kinetics of the appearance of the ingested glucose in the bloodstream depends on the characteristics of the ingested starch. At the same time, insulin is secreted by the pancreas decreasing EGP and increasing glucose utilisation by the tissues.

As a consequence, a moderate postprandial glucose response may not only indicate a slow appearance of ingested CHOs and slow tissue uptake but could also result from accelerated appearance and tissue uptake of the ingested CHOs. This highlights the necessity of describing the overall metabolic response kinetics to CHO ingestion rather than simply the glycaemic profile, which combines the difference between incoming and outgoing glucose flow rates. Stable glucose isotopes can be used to follow the kinetics of exogenous glucose from the ingested meal and EGP, and to identify the mechanisms underlying variations in postprandial glycaemia. The isotopic double labelling technique can differentiate between glucose from food (using ^13^C-labelled starch) and whole-body glucose (using a deuterated glucose infusion) to trace total body glucose. For 20 years, our team has pioneered the use of this double labelling technique to investigate the fate of ingested CHO (absorption, uptake, and oxidation) (31, 32). The measurement of plasma ^13^C-glucose enrichment by mass spectrometry coupled with gas chromatography, together with mathematical modelling, is used to measure the rate at which exogenous glucose appears in the plasma, in order to follow the postprandial kinetics of ingested starch. Total glycaemia and EGP are also investigated.

**Fig. 3:**
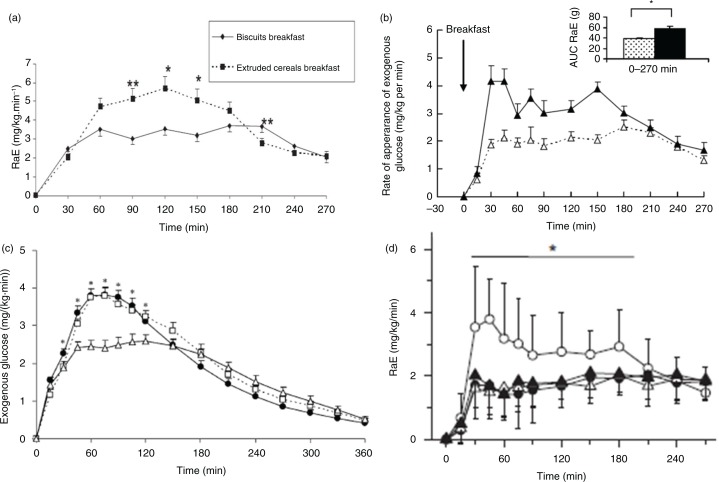
An increased slowly digestible starch content slows the appearance of exogenous glucose in the bloodstream. AUC, area under the curve; HGI, high glycaemic index; LGI, low glycemic index; RaE, rate of appearance of exogenous glucose; SDS, slowly digestible starch. (a) LGI/high-SDS biscuits (23%) (◆) versus HGI/low-SDS extruded cereals (1.5%) (■) (from Vinoy et al. (27)); (b) LGI/high-SDS biscuits (26%) (Δ) versus HGI/low-SDS extruded cereals (<1.0%) (▲) (from Nazare et al. (32)); (c) SDS pasta (10.6%) (Δ) versus control bread (6.1%) (●) versus purple bread (5.4%) (□) (from Eelderink et al. (7)); (d) Three biscuits (39–45% SDS: high-SDS breakfast) (●,△,▲) versus extruded cereal (0.3% SDS: low-SDS breakfast) (○) (from Péronnet et al. (33)).

By applying these analytical methodologies to clinical interventions, several groups have shown that foods containing a high level of SDS induce a slower glucose appearance compared with foods with a low-SDS content (7, 27, 32, 33) (Fig. 3).

Depending on intrinsic or extrinsic factors (such as processing or cooking), starch exists in three forms, namely RS, RDS, and SDS. It has been shown that a breakfast with cereal foods high in SDS significantly reduces the appearance of exogenous glucose in the early part of the morning and extends its release into the later part of the morning (27). These results were confirmed in a study of 38 overweight subjects who ingested a low-GI and high-SDS breakfast or a high-GI and low-SDS breakfast for 5 weeks (32). Breakfast SDS content appears to be an important parameter for metabolic control of the glycaemic response throughout the morning.

**Fig. 4:**
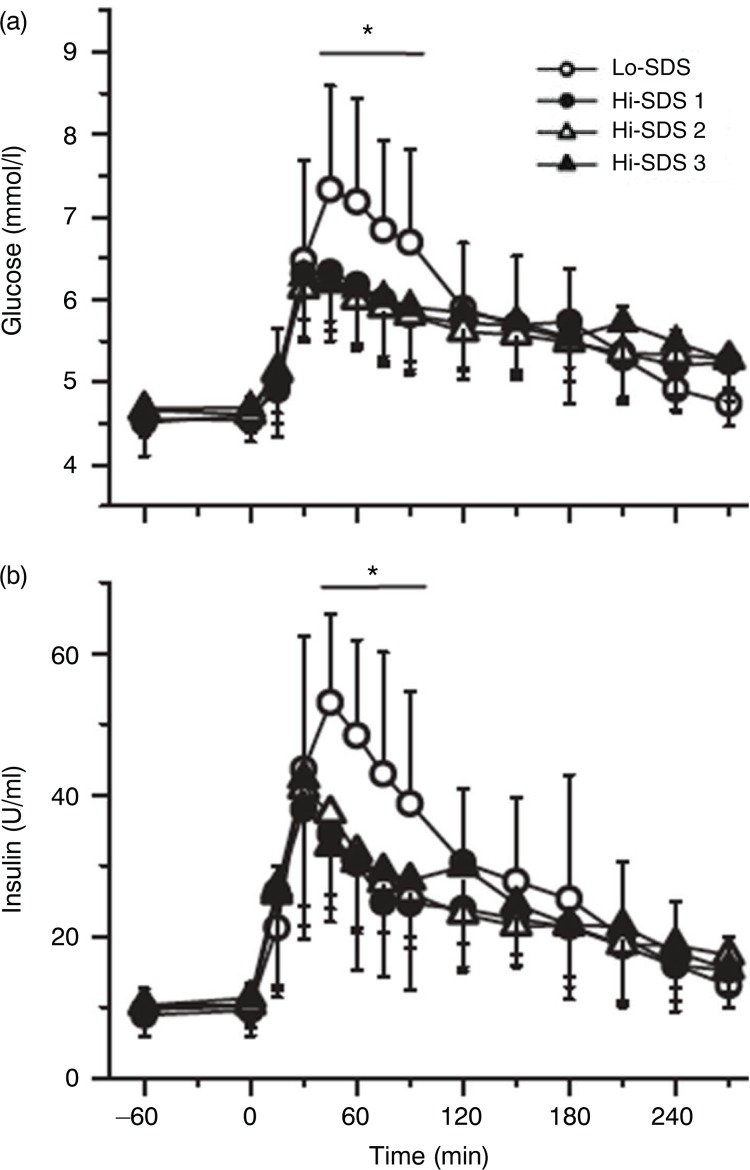
Plasma glucose (a) and insulin (b) following the ingestion of breakfast with a high or low-SDS content. Hi-SDS, high-SDS content; Lo-SDS, low-SDS content. From Péronnet et al. (33). *P<0.05: significantly different from the Hi-SDS breakfasts.

A glucose kinetics analysis by Peronnet et al. (33) provided an explanation for the regulatory mechanisms underlying changes in glycaemia: foods with a high-SDS content induced a slower rate of glucose appearance from starch (Fig. 3d) with lower insulin secretion (Fig. 4b), which in turn led to lower compensatory inhibition of endogenous hepatic glucose production. Thus, the global reduction in glycaemia (Fig. 4a) was less significant than expected based on observed differences in the appearance of exogenous glucose (33).

Moreover, the glycaemic response to the subsequent standardised lunch was also reduced in the group that consumed a high-SDS breakfast, suggesting that modulation of the glucose availability at breakfast was a determinant for glucose tolerance throughout the day. Eelderink et al. (7) observed that in healthy subjects, the reduced rate of glucose appearance resulted in a lower gastric inhibitory polypeptide (GIP) concentration and lower insulin response, but similar postprandial glycaemic response, following the ingestion of high-SDS pasta, compared with bread. This highlights the fact that other underlying metabolic processes occur simultaneously to counteract the slower rate of absorption. The prolonged glucose release after SDS ingestion may induce a late postprandial elevation in gut hormones (GIP and GLP-1), which could have interesting physiological consequences on satiety and gastric emptying in relation to the second meal effect (34). These results also highlight the potential beneficial effect of SDS on insulin sensitivity by reducing the demand for insulin production from the pancreas (7). In overweight or obese subjects, SDS may also indirectly affect insulin sensitivity by improving the fasting (35) or postprandial lipid profile (36).

In conclusion, consumption of high-SDS food is associated with lower postprandial glycaemia due to slower glucose release, lower postprandial insulinaemia, and stimulation of gut hormones, all of which have potential health benefits. To understand the metabolic effects of CHOs on metabolic control throughout the day and on the cardiometabolic risk profile, it is essential to monitor the overall glucose kinetics as well as hormonal and lipid responses in the postprandial phase.

### Interest of reducing postprandial glycaemic response in the prevention of metabolic diseases

Prof. Edith J.M. Feskens, Wageningen University

Metabolic diseases such as metabolic syndrome (MetS) are highly prevalent in Western societies. MetS is not actually a disease but describes a group of risk factors underlying cardiovascular and metabolic disease (i.e. cardiometabolic diseases), including T2DM (37). The increase in MetS incidence is largely due to the well-known increase in abdominal obesity, and it is expected that the number of cases worldwide will continue to increase dramatically in keeping with the trends of overweight and obesity (38).

In daily practice, with regard to clinical investigations concerning lipids or glucose, patients are asked to fast. This makes sense, as meals induce a metabolic response and standardisation is important. However, for most of the day, people are not in the fasting state but fed, and hence in some sort of postprandial stage. With the recent introduction of continuous glucose monitoring systems, we have gained additional information regarding the daily fluctuations in glucose levels. So far, continuous glucose monitoring systems have not yet been used in follow-up studies but interesting differences in daytime interstitial glucose levels, postprandial glucose excursions, and postprandial peaks have been observed in subjects with normal to moderate hyperglycaemia (39).

**Fig. 5:**
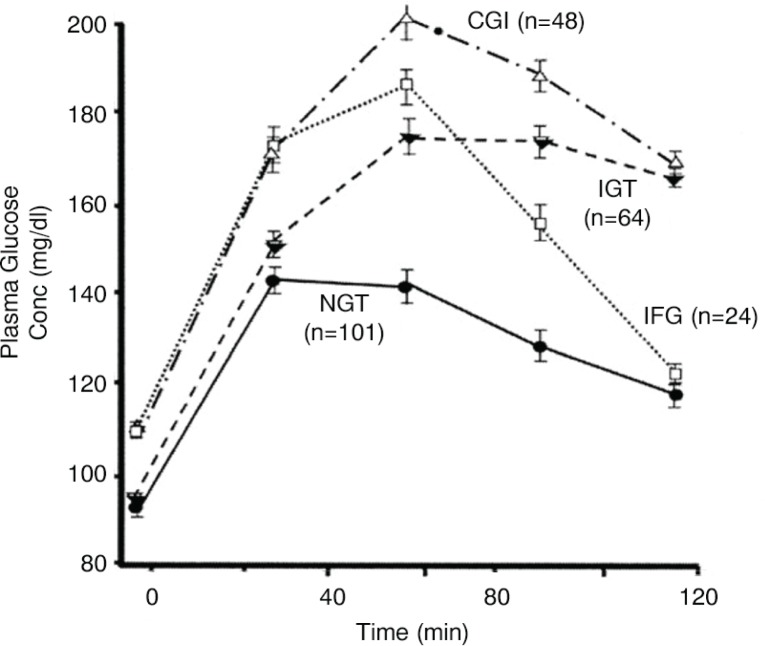
Plasma glucose concentrations during oral glucose tolerance test performed in subjects with normal glucose tolerance, impaired fasting glucose or glucose tolerance, and combined glucose intolerance. CGI, combined glucose intolerance; IFG, impaired fasting glucose; IGT, impaired glucose tolerance; NGT, normal glucose tolerance. From Abdul-Ghani et al. (41).

In the diabetes field, the importance of the postprandial period has been recognised since long, and oral glucose tolerance tests (OGTTs) have been used to detect diabetes using a combination of elevated fasting and 2-h glucose levels (after ingestion of a 75 g glucose load) (40). The OGTT is used in population-based surveys but is seldom used in everyday practice. Elevated fasting levels are generally due to hepatic glucose production, while postprandial levels are mainly affected by reduced glucose uptake by tissues such as muscle and can exhibit elevated levels before overt diabetes is detected. This is particularly relevant for preventing cardiometabolic disease, as insulin resistance is an important underlying pathogenic factor. Intermediate stages between normoglycaemia and T2DM can be identified due to their different response profiles:

- Impaired glucose tolerance (IGT) is associated with elevated 2-h glucose levels and the presence of muscle insulin resistance.
- Impaired fasting glucose (IFG) is associated with elevated fasting levels only, as well as the presence of hepatic insulin resistance (41) (Fig. 5).

It should be noted that the original cut-off points for diabetes were derived from the Pima Indians, a Native American group with very high rates of obesity and diabetes (40). A study of Pima Indians showed that 2-h glucose levels predicted the risk of diabetes, similar to glucose fasting levels. However, this was especially clear at the higher end of the glucose distribution, which is typical for such a high-risk group (42).

The importance of normoglycaemic 2-h glucose levels was investigated by Abdul-Ghani et al. (43) as part of the San Antonio Heart Study. This group studied the progression to T2DM based on fasting versus 2-h post-load glucose levels during 7–8 years of follow-up in subjects who were free from diabetes, IGT, and IFG at baseline. In 23 subjects (group I), the plasma glucose concentration recorded during the OGTT returned to levels below the fasting concentration at 30 min, whereas in 111 subjects (group II) and 313 subjects (group III), the plasma glucose concentration recorded during the OGTT returned to levels below the fasting concentration at 60 and 120 min, respectively. In the remaining subjects (*n*=835, group IV), the plasma glucose concentration recorded during the OGTT never fell below the fasting concentration. The results showed that subjects whose post-load plasma glucose concentration returned to fasting level more quickly had greater insulin sensitivity, a higher insulinogenic index, and a lower risk of developing T2DM after 8 years of follow-up compared with subjects whose post-load glucose concentration returned to baseline more slowly. The incidence of T2DM was 0% in group I and increased progressively to 6.4% in group IV.

Ning et al. (44) performed a similar study to investigate the association between fasting, 2-h glucose levels and the risk of cardiovascular disease (CVD). This study used data from 19 European cohorts that included 12,566 men and 10,874 women with normal fasting plasma glucose (FPG) and 2-h glucose levels. The study population was divided into two groups: those with a 2-h plasma glucose level that was higher than the fasting concentration (group II) and those with a 2-h plasma glucose level that was lower than or equal to the fasting concentration (group I). Having a high 2-h plasma glucose level compared with the fasting concentration (group II) was associated with insulin resistance and higher mortality from CVD. After adjusting for age, FPG, BMI, total cholesterol, and fasting insulin, the relative risk of death due to CVD was 1.25 in men and 1.60 in women. Both men and women in this group had an 18% increased risk of all-cause mortality. Both of these elegant studies thus showed that, within the normal range, having an elevated 2-h glucose level after ingesting a 75-g glucose load is predictive of T2DM, CVD, and all-cause mortality. The potential underlying mechanisms for this postprandial risk have been reviewed by Standl et al. (45). Increased glucose auto-oxidation, disordered endothelial function, increased low-grade inflammation, increased blood coagulation, reduced fibrinolysis, decreased plaque stability, reduced triglyceride-rich lipoprotein and LDL removal, increased HDL cholesterol catabolism, reductions in free fatty acids, reduced early phase insulin secretion, and increased insulin resistance are all factors that are thought to play a role.

**Fig. 6:**
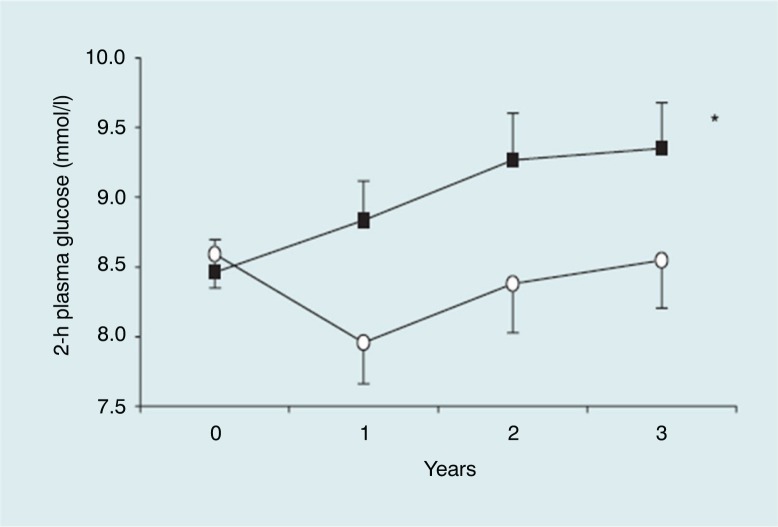
Two-hour plasma glucose levels for the intervention and control groups at baseline and after 1, 2, and 3 years of follow-up. (○) intervention group, (■) control group. Data are means±SEM, *n*=106 (52 in intervention group, 54 in control group). * Indicates a significant difference between groups over time, p=0.023 (general linear model ANOVA for repeated measures). From Roumen et al. (46).

In addition to observational studies, intervention studies have also demonstrated the importance of postprandial glucose levels. An example is our own SLIM study, which showed that a combined lifestyle intervention based on a healthy diet and increased physical activity reduced 2-h glucose levels over the 3-year study period (46) (Fig. 6). This intervention was similar to that implemented by the Diabetes Prevention Study (DPS) (47), and also reduced the incidence of diabetes by 50%, similar to results from the DPS and Diabetes Prevention Programme (48).

In conclusion, postprandial hyperglycaemia is an independent risk factor for T2DM and CVD, and reducing 2-h plasma glucose levels in prediabetics is associated with a decrease in conversion to T2DM. As plausible aetiologic mechanisms for the relationship between postprandial glycaemia and disease development exist, we argue that postprandial glucose levels are relevant for health and disease and represent a meaningful target for intervention, for example, through modifying dietary factors.

## Discussion

The forum session clarified the importance of SDS in regulating the postmeal metabolic response. By controlling food-processing conditions to limit starch gelatinisation, cereal products with a high-SDS content induced a slower CHO appearance rate, which led to lower glycaemic and insulin responses compared with cereal products that contained virtually no SDS. In the short term, elevated blood glucose levels and an elevated insulin concentration lead to a transitory deleterious metabolic state, involving the liver, lipid metabolism interactions, and incretins, in both healthy and glucose-intolerant subjects. Conversely, short-term clinical studies show that a decrease in the postprandial glycaemic response combined with a non-exacerbated insulin demand could be beneficial for health maintenance and help reduce the risk of metabolic disease.

Elevated postprandial glycaemia and related insulinaemia and lipaemia have been implicated in the aetiology of chronic metabolic diseases such as T2DM and CVD (2). In addition, epidemiological studies have identified an independent positive relationship between blood glucose levels and the development of metabolic disease (49). These data can be cautiously extrapolated to long-term disease prevention. Several medium- to long-term intervention studies that compared low-GI diet and high-GI diet have been published in the last 20 years (3). However, the majority of these human studies did not measure differences in the postprandial glycaemic and insulin responses to the high-GI diet versus the low-GI diet. In general, the authors based their assumption on GI values calculated from GI tables. Unfortunately, the calculated GI and the measured GI of meals/diets can vary dramatically (50). In fact, the calculated GI does not take into account the interactions between macronutrients. It was recently shown that some macronutrients (SDS, fat, and fibres) and their interactions can have a strong influence on glycaemic response (29). Thus, while there is some evidence that replacing high-GI foods with low-GI foods may help decrease the risk of metabolic disease, it has not been possible until now to quantify the extent to which the low glycaemic response associated with a lower insulin response is beneficial. This is primarily due to the lack of well-designed long-term studies that are able to quantify the reduction in glycaemic and insulin responses in human subjects. Analysing the continuously measured glucose profiles of non-diabetic subjects has already yielded interesting information regarding the duration of the postprandial state over a 24-h period (39). This method can be used over several days to quantify the blood glucose levels at home during the intervention period.

Some long-term intervention studies provide data from OGTT measurements. The OGTT is a challenge test that evaluates the body's response to a large quantity of glucose. This is a gold standard method for evaluating glucose intolerance and can help to diagnose T2DM. The hypothesis of research is to show an improvement in OGTT parameters after subjects consumed a low-GI diet compared with a high-GI diet over a long-term intervention period, because the subjects’ metabolic status improved due to a decrease in the postprandial glycaemic response (51). The limitation of these study designs is that the postprandial glycaemic response is not measured. Thus, there is a clear need to design long-term studies that quantify the postmeal glycaemic response, in order to validate the design of the intervention (either low or high glycaemic response diets), and measure the OGTT, to quantify the metabolic status of the subjects and any potential improvement due to the intervention. Both of these complementary methods are very useful in intervention studies.

Another parameter needs to be addressed regarding the low glycaemic response and its health benefits. As mentioned during the symposium, foods with a high-SDS content can reduce glycaemic and insulin responses compared with products that contain only RDS, due to the slow release of CHOs during the postprandial phase. In several studies that manipulated GI, the way to decrease the GI was not mentioned, much less studied or debated. In fact, there are several digestive and/or metabolic ways for decreasing the GI or the glycaemic response in a food or diet. A recent review reported that some components significantly decrease the glycaemic response to CHO-rich foods (20). These data show that viscous soluble fibres, SDS, lipids, and fructose decrease the GI or glycaemic response without exacerbating the insulin demand. In contrast, some types of protein dramatically exacerbate the insulin response, which results in a decrease in the GI. This increase in the insulin response stimulates blood glucose uptake by the peripheral tissues. It is possible that not all of these ways have the same metabolic consequences in the long term and this should be debated. For example, SDS and viscous soluble fibres can effectively decrease the GI and may be associated with long-term health benefits, whereas fat or fructose should only be used in limited amounts to lower the glycaemic response to products or meals, as they may not have any additional health benefit (20).

Thus, there is a clear need to better characterise tested products, specifically in terms of their SDS/RDS contents as well as the content of other macronutrients (digestible and non-digestible) to better understand the health-related effects of low and high glycaemic response diets. Establishing the SDS content of foods and diets used in metabolic studies may clarify the usefulness of lowering the glycaemic response in disease prevention.

### Key messages

Starch represents the largest component of daily energy intake in humans. Consumption of starchy foods leads to a transient postprandial glycaemic response, and the profile of this response varies widely according to the sensitivity of starch to digestive amylases. In this symposium, the latest findings on the relationships between slow-release CHOs, the glycaemic response, and public health were presented. The key points are as follows:

1. A method validated *in vitro* that measures the rate and extent of starch digestion is available. This is the method developed by Englyst et al. (25). It classifies starch into three fractions: RDS, SDS, and RS.
2. In its native (raw) state, starch is digested slowly. SDS can be preserved in starchy processed foods by selecting appropriate ingredients that are rich in SDS and by controlling processing conditions to limit starch gelatinisation.
3. It has been consistently shown in humans that high-SDS foods decrease the glycaemic response.
4. Use of the double labelling technique provides information regarding the underlying mechanism of how SDS content decreases the glycaemic response: high-SDS foods slow the appearance of exogenous glucose in plasma, leading to a decrease in the acute glycaemic response through the decrease in insulin secretion, stimulation of gut hormones, and a less inhibited EGP, compared with foods with virtually no SDS.
5. SDS-rich cereal products improve fasting and postprandial lipid metabolism in overweight and dyslipidaemic subjects.
6. Postprandial hyperglycaemia is an independent risk factor for T2DM and CVD.
7. There are some examples of combined lifestyle interventions based on a healthy diet and increased physical activity that were able to reduce 2-h glucose levels (as measured by the OGTT) over several years.
8. More well-designed, long-term studies are needed to investigate the potential beneficial effects of decreasing postprandial glycaemic responses via the consumption of high-SDS foods on insulin sensitivity and the cardiometabolic risk profile.
